# Towards translation of environmental determinants of physical activity in children into multi-sector policy measures: study design of a Dutch project

**DOI:** 10.1186/1471-2458-9-396

**Published:** 2009-10-27

**Authors:** Marie-Jeanne Aarts, Ien AM van de Goor, Hans AM van Oers, Albertine J Schuit

**Affiliations:** 1 Tilburg University, Faculty of Social and Behavioural Sciences, Department Tranzo, PO Box 90153, 5000 LE Tilburg, The Netherlands; 2 National Institute for Public Health and the Environment, Public Health and Health Services Division, Centre for Public Health Forecasting, PO Box 1, 3720 BA Bilthoven, The Netherlands; 3 World Health Organization, Avenue Appia 20, 1211 Geneva 27, Switzerland; 4 VU University Amsterdam, Faculty of Earth and Life Sciences, Department of Health Sciences, De Boelelaan 1105, 1081 HV Amsterdam, The Netherlands

## Abstract

**Background:**

Physical inactivity in children is a major health problem in The Netherlands as well as in many other Western countries. In addition to health promotion among parents and children, creating "active" neighbourhoods can contribute to the solution of this health problem. However, changing environmental characteristics is often the responsibility of policy sectors outside the Public Health domain. Therefore this project identifies and evaluates the possibilities of multi-sector policy measures to stimulate physical activity in children.

**Methods and design:**

The project consists of quantitative as well as qualitative research methods and is conducted in four medium sized Dutch cities. To identify perceived environmental determinants of physical activity in children, a large scale health survey was conducted at 42 primary schools. Written questionnaires including topics on the children's physical activity behaviour (i.e. sports participation, outdoor play, active commuting, television watching and computer usage) and physical and social environmental characteristics were completed by 6,601 parents of children aged 3-13 years old and 3449 children aged 9-13 years old. In addition, 33 neighbourhood audits (systematic observations) were conducted to assess objective neighbourhood characteristics. Furthermore, a policy analysis was conducted in the four participating municipalities to provide an overview of the current local policy measures directed at stimulation of physical activity in children. Policy plans of six different policy sectors (Public Health, Sports, Education & Youth, Spatial Planning, Traffic & Transport, and Safety) were screened for their content on physical activity in children. In addition, semi-structured interviews were conducted with policy makers of each of these sectors to identify critical success factors in the development and realization of multi-sector policy plans aimed at stimulating physical activity in children. The results of all these research activities will be discussed with local policy makers during interactive workshop sessions in order to identify clear cut multi-sector policy measures that stimulate physical activity in children.

**Discussion:**

This paper describes the study design of a project that focuses on multi-sector policy measures that stimulate physical activity in children. Next to extensive research into the environmental determinants of physical activity in children, much emphasis is placed on the translation of the research outcomes into concrete and feasible policy plans.

## Background

### Lack of physical activity among youth: the role of the environment

Although The Netherlands is famous for its pedestrian and bicycle friendly infrastructure, lack of physical activity is a serious problem among the Dutch population. Not only is approximately 50% of all Dutch adults not as physically active as would be desirable for their health [1], also a great percentage of children is relatively inactive. Only a quarter of the Dutch primary school children meets the recommended guidelines for physical activity as stated by the Dutch health authorities, which encompasses at least 60 minutes of moderate physical activity for at least 5 days per week [2]. One study conducted in deprived Dutch neighbourhoods even showed that only 3% of the children who lived there met the recommended guidelines [3]. Hence, the vast majority of the Dutch children is not sufficiently involved in regular physical activity, particularly those living in deprived areas.

Besides the increased risk of developing overweight and obesity, an inactive lifestyle during youth increases the risk for the development of cardiovascular disease, hypertension, diabetes, psychosocial problems and a poor development of motor skills [4,5]. Particularly when the inactivity is maintained on a lifetime basis, the health consequences may be severe. It is therefore of major importance to find appropriate ways to stimulate physical activity in children.

Until recently, most Dutch prevention initiatives have focussed on health education among children and their parents, often in a school based setting. Several teaching programs and other school-based physical activity programs for primary and secondary schools have been developed to create awareness among teachers, parents and children for the benefits of an active lifestyle and for the opportunities to be physically active. Some of these interventions are based on behavioural change theories, take into account the school environment and have been evaluated on their effectiveness in getting children more physically active [6-8].

Nowadays, progressively more attention is drawn to the influences of environmental characteristics on children's physical activity level. Numerous studies addressing the role of physical as well as social environmental determinants in physical activity behaviour of adults and youth are conducted in North America [9-14] or Australia [15-17]. An overview of potential environmental determinants of physical activity specific for youth is given recently by Ferreira et al. [18]. Next to potential environmental determinants at the home level and at the school level this review includes a limited number of potential determinants at the neighbourhood level. In contrast with some home and school level characteristics such as father's physical activity level and school physical activity policy, there was no clear relationship found between physical and social neighbourhood characteristics and children's physical activity level. For example access to or availability of physical activity facilities or programs, neighbourhood hazards (such as dangerous traffic situations), and social safety were consistently unrelated to children's physical activity. Due to the limited number of studies addressing other neighbourhood characteristics such as distance to destinations, available shelter/food path conditions, neighbourhood physical disorder and neighbourhood social disorder, no conclusions could be drawn regarding their relationship with physical activity. These findings emphasise the need for more research into the influence of neighbourhood characteristics on children's physical activity level.

Moreover, only 13.5% of the studies that were included in the abovementioned review were situated in Europe. Because the European environmental setting may differ drastically from that in North America or Australia (e.g. in terms of street pattern, traffic situation, sports and play facilities, social or societal structure, etc.) it is of great importance to gain more insight in potential environmental determinants of physical activity in children at a national or even local level.

It can be concluded that lack of physical activity among children is a complex problem which needs an integrative approach, aimed at individual as well as environmental determinants [19]. This research project will focus on the environmental determinants of physical activity and the role of local policy measures herein, which will be expounded below.

### The need for evidence based multi-sector policy actions

The importance of environmental determinants of healthy behaviour in general, and more specifically physical activity among children, is recognised by the national government in The Netherlands. In the National Memorandum on Overweight from the Ministry of Health, Welfare and Sport [20] it is stated that a healthy local environment is essential for long-lasting successful prevention of obesity. According to the Memorandum, local governments (such as municipalities), play a crucial role in creating a healthy environment i.e. "making the healthy choice the easy choice".

Effectively addressing physical as well as social environmental determinants of physical activity in children, is for a large part dependent on policy measures outside the Public Health domain. For example Sports, Education & Youth, Spatial Planning, Traffic & Transport and Safety can all contribute to a more activity-friendly environment for children [21,22]. Working across sectors towards a coherent policy plan for stimulating physical activity in children, is therefore considered necessary. In The Netherlands, most municipalities do recognise the benefits of such an approach, but local policy makers still find it challenging to develop and implement such multi-sector policy plans. Information about success and failure factors in multi-sector collaboration is particularly found in so-called "grey literature" [23]. Effectiveness of multi-sector policies is hard to measure [24] and is for instance dependent on which sector takes the initiative, the point in time or policy process in which the collaboration is started, the basis of support for the multi-sector initiative, the amount of resources and manpower available, the existence of shared (policy) goals and the availability of (scientific) information regarding the potential effectiveness of multi-sector policies [25]. Multi-sector policy plans are more effective when the actors involved share common interests and conflicts of interests are absent. The presence of key figures or "policy entrepreneurs" and structural and long term multi-sector collaboration can also increase the potential success of multi-sector policies [26].

Little scientific publications however are available about success and failure factors in multi-sector policy development and implementation. Better understanding of success factors and barriers in multi-sector policy development can contribute to an integral and long term approach in tackling physical inactivity in youth.

Although highly potent in improving children's physical activity level in large populations, redesigning neighbourhoods and structurally improving opportunities and facilities for active living, can be very costly. It is therefore utterly important to have scientific evidence underpinning possible multi-sector policy measures, i.e. to develop evidence based policy plans on the local level. Several studies however show that scientific knowledge only plays a modest role in the policy development process and governmental decision making [27]. This project therefore specifically focuses on the translation of scientific research results into feasible and concrete multi-sector policy measures.

### Aim of the study

The aim of the project is to identify, describe and test the feasibility of concrete multi-sector policy measures to stimulate physical activity in children in a concerted action between researchers and policy makers. The project consists of four major parts:

1) A health survey among primary school children and their parents to identify perceived environmental determinants of physical activity in children;

2) Neighbourhood audits to identify objective environmental determinants of physical activity in children by means of systematic observations;

3) Policy analysis of the current local policy situation regarding environmental determinants of physical activity in children in four municipalities to identify promising possibilities and critical success factors for a multi-sector approach;

4) Interactive workshop session with local policy makers to identify clear cut and feasible multi-sector policy measures that stimulate physical activity in children.

The goal of the first 3 parts is to identify the environmental determinants of children's physical activity behaviour and to map the current policy situation. The last part of the project is aimed at translation of the data collected in the first three parts into concrete multi-sector policy actions at the local level. The ultimate goal of the research project is to provide scientific support for local policy makers in developing multi-sector policy measures to stimulate physical activity in children. Quantitative as well as qualitative methods are combined in this project and the different parts of the project will be described in more detail below.

## Methods/study design

### Study setting

At the start of the project in October 2006, five municipalities were approached for participation in the project by letter and were given more detailed information during a personal meeting. The municipalities were selected based on their similarities in population size and composition. Moreover, these cities were chosen from the service domain of the Regional Public Health Services which in turn are cooperating in the Academic Collaborative Centre Public Health of Tilburg University. Academic Collaborative Centres are proposed to contribute to bridging the gap between science, practice and policy in public health [28]. The Regional Public Health Services are the regular advisors of municipalities in public health affairs and their expertise and contacts in the field were utilized to recruit the municipalities for the research project. Due to lack of time and interest in the topic, one municipality decided not to participate. Hence, the research project is conducted in four medium sized cities in The Netherlands: Tilburg, Breda, 's Hertogenbosch and Roosendaal. Although the project was not initiated by the municipalities themselves, they declared to be interested in the topic and willing to cooperate in the project. No financial or other obligations or compensations were asked from or given to the municipalities in order to participate in the project.

Table 1 summarises the main characteristics of the four cities that were enrolled in the study. This table indicates that, although there are some differences between the cities, the four municipalities show much resemblance regarding size and composition of their population.

**Table 1:** Population characteristics of municipalities included in the study*

	**Tilburg**	**Breda**	**'s Hertogenbosch**	**Roosendaal**
Total number of inhabitants	201,259	170,349	135,648	77,450
Degree of urbanisation (number of inhabitants per km^2^)	1,716	1,344	1,606	727
Percentage of inhabitants aged 0-14 years (%)	16.7	17.3	17.2	17.6
Percentage western immigrants** (%)	8.2	10.0	8.6	8.7
Percentage non-western immigrants*** (%)	13.4	10.2	9.9	11.9

* Characteristics per 01-01-2007

** Immigrants are defined as persons with at least one parent born in a foreign country. Western immigrants are all immigrants from Europe (with exception of Turkey), North-America, Oceania or Indonesia or Japan.

*** Immigrants are defined as persons with at least one parent born in a foreign country. Non-western immigrants are all immigrants from Turkey, Africa, Latin-America or Asia (with exception of Indonesia and Japan).

All data derived from CBS Statline [2].

### Target population

The project is targeted at primary school children age 3-13. The influence of environmental characteristics may be especially important for younger children, who have less autonomy to travel long distances by themselves and therefore are more dependent on their direct environment [29,30]. Secondly, because childhood obesity is shown to track into adulthood [31] the benefits of an active life style are greater when physical activity is introduced at an early age and is maintained throughout the entire life span.

### Health survey

To identify the determinants of physical activity in children, a large scale health survey was conducted among primary school children (age 3-13) and their parents. With exception of those schools that were known to be already participating in another (research) project aimed at physical activity in children (n = 34), all regular primary schools (n = 149) of the four municipalities were invited by letter and thereupon by telephone to participate in the survey. Approximately one third of all invited schools (n = 42) agreed on participation. Table 2 shows the distribution of the participating schools among the four cities and the average number of pupils per school. Average school size was 255 pupils per school, which was somewhat higher in Tilburg due to inclusion of 2 schools with more than 500 pupils.

**Table 2:** School recruitment and school size in the four participating cities

	**Tilburg**	**Breda**	**'s Hertogenbosch**	**Roosendaal**	**Total**
Total number of school	49	35	43	22	149
Number of schools included in the survey	13	13	8	8	42
Percentage of schools included in the survey	26.5%	37.1%	18.6%	36.4%	28.2%
Average school size of schools enrolled in the survey (number of pupils)	310	252	248	231	255

The schools were scattered geographically among the four cities, varying in location from mid-centre to the periphery. As indicator for physical (or "built") environment, a measure of the Ministry of Housing, Spatial Planning and the Environment was used, which categorises postal code areas as either "city centre", "city non-centre", "city green", "town centre", "rural area" or "work area" [32]. From table 3 it can be concluded that our study sample is a good reflection of the total population of schools in the four cities. Although more than 50% of the included schools are classified as "city non-centre", this does not imply that these neighbourhoods are very similar with regard to their physical environmental characteristics. In fact, there are large differences in for example, type of buildings, traffic situation and sports facilities.

**Table 3:** School's characteristics: built environment

	**Number of participating schools**	**Percentage (%)**	**Total number of schools**	**Percentage (%)**
City centre	3	7.1	10	6.7
City non-centre	24	57.1	82	55.0
City green	14	33.3	53	35.6
Town centre	0	0.0	0	0.0
Rural area	1	2.4	4	2.7
Work area	0	0.0	0	0.0

Total	42	100.0	149	100.0

As an indicator for social environment, we used a measure from The Netherlands Institute for Social Research which is called "status score" and is based on percentage immigrants, percentage people with low education and percentage low income households per postal code area [33]. Low status scores correspond with high socio-economic status. As shown in table 4, there is great variety in socio-economic status of the schools' neighbourhoods, as indicated by status score. Schools from lower socio-economic neighbourhoods (as indicated by a high status score) were somewhat underrepresented in our study sample, because these schools were relatively often involved in other projects aimed at promotion of physical activity in children, and therefore less willing to participate. Nonetheless, we succeeded to include almost 10% of the total number of schools in the lower socio-economic neighbourhoods in our health survey.

**Table 4:** School's characteristics: socio-economic status*

	**Number of participating schools**	**Percentage (%)**	**Total number of schools**	**Percentage (%)**
School with status score < -2	5	11.9	14	9.4
Schools with status score ranging from -2 till -1	10	23.8	32	21.5
School with status score ranging from -1 till 1	12	28.6	40	26.8
Schools with status score ranging from 1 till 2	11	26.2	40	26.8
Schools with status score > 2	4	9.5	23	15.4

Total	42	100.0	149	100.0

* High status score values represent low socio-economic status

**Figure 1:**
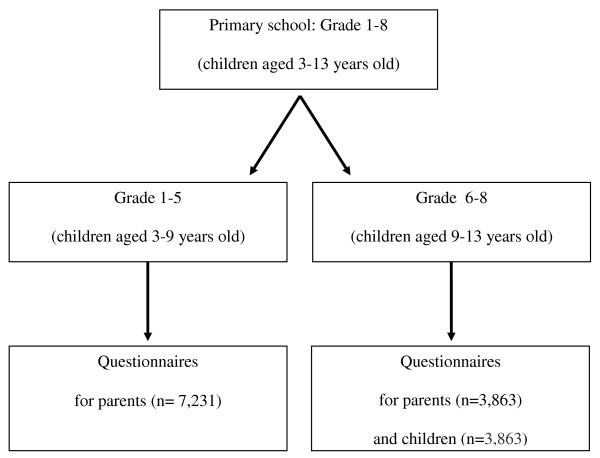
**Survey design at primary schools**.

At each school willing to participate, all grades and classes were included in the study. As indicated in figure 1, the children of all grades (grade 1 to 8, corresponding with age 3-13 years old) were given a questionnaire to take home for their parents. The children of the highest grades (grade 6, 7 and 8, corresponding with age 9-13 years old) were also asked to fill in a questionnaire themselves during class hours.

The health survey was conducted between September 2007 and January 2008. In total, 3,863 children (age 9-13 years old) and 11,094 parents of children aged 3-13 years old of 42 schools, were asked to fill out a questionnaire. Assuming a response rate of 50% and ICC = 0.06 representing the clustering of the data within schools, the effective sample sizes reduce to 522 children and 626 parents [34]. The power to detect a small effect *f*^2 ^= 0.02 in a multiple regression analysis at *α *= 0.05 with 35 predictors, given these sample sizes, equals 0.90 and 0.94 for the children's and parent's data, respectively. The power analyses were performed using G*Power 3 [35].

Because no medical or physical measurements were conducted and considering the negligible (psychological) burden for children to fill in the questionnaire, no ethics approval was required (according to the Dutch Central Committee on Research investigating Human Subjects). Children were given written and verbal information about the survey in class and were free to renounce from participation without giving any reason. Parents were given written information about the study and by returning the completed questionnaires, parents gave their consent for inclusion of their data in the data base. Parents were offered the opportunity to object to the inclusion of their child's questionnaire as well by a pre-printed objection letter. Parents of 71 children objected against the inclusion of their child's questionnaire in the data base, in those cases the child's questionnaire was destructed. All questionnaires were distributed and collected completely anonymous.

Separate questionnaires were developed for parents and children. The questionnaires were based on questionnaires that were used in other large scale research projects in the Netherlands (results not yet published) but refined to fit the specific research questions of this project. The questionnaires were pre-tested in a pilot sample of parents and children which lead to some small adjustments with respect to the formulation of the questions.

The questionnaire for the parents included questions on the child's physical activity habits (e.g. sports participation, active commuting to and from school, outdoor play and inactive leisure time activities such as television watching and computer usage), topics on the physical environment (e.g. outdoor play opportunities, sports facilities, distance to other facilities, public space design, traffic safety, street pattern, type of buildings) as well as the social environment (e.g. social cohesion, area deprivation, social safety, financial barriers for sports participation). In addition to these environmental characteristics there were also questions about the home environment (household composition, family customs and norms, number of electronic devices and cars in household, support by parents/siblings/peers). Individual factors (such as income, education level and work situation of the parents, ethnicity, age, gender and body mass index (BMI) of parents and children and overall health of the child) as well as some additional questions about eating and sleeping habits were also included.

The questionnaire for the children covered roughly the same topics, but was less elaborate and did not include questions on the socio-economic status of the parents. Especially for older children, who are less parent dependent for their mobility, the views and opinions about environmental characteristics that enhance or hamper physical activity may deviate from their parent's viewpoints. By administering questionnaires among both parents and children, the perception of the neighbourhood characteristics of parents can be compared with that of their children. To prevent parental influences on the answers given, children completed the questionnaire during school time.

Finally, the survey also included a questionnaire that was filled in by the management of each of the 42 participating schools. This questionnaire included topics on the physical education program at school, the physical activity policy program, schoolyard play opportunities and traffic situation around the school. This questionnaire will be used to assess school environmental characteristics and school policies related to children's physical activity level.

**Figure 2:**
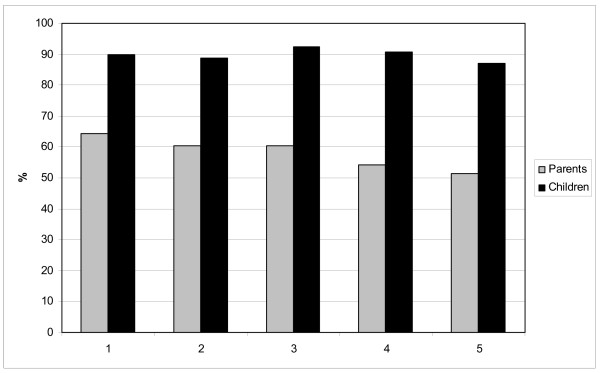
**Response rates of parents and children on questionnaires by school's socio-economic status**. 1 = Schools with status score < -2 *. 2 = Schools with status score ranging from -2 till -1 *. 3 = Schools with status score ranging from -1 till 1 *. 4 = Schools with status score ranging from 1 till 2 *. 5 = Schools with status score > 2 *. * Low status score represents high socio-economic status.

The overall response on the questionnaires was high: on average 60% of the parents completed and returned the questionnaire and 90% of the children completed the questionnaire. Also, the management of each participating school completed the questionnaire. Figure 2 shows that the response of parents was somewhat lower in the lower socio-economic schools, most probably partly due to language difficulties.

### Neighbourhood audits

In addition to the perceived environmental characteristics as measured in the health survey, neighbourhood characteristics were also objectively measured by means of neighbourhood audits consisting of standardized observations. Previous studies show that perceived environmental characteristics can deviate from objectively measured environmental characteristics [9]. It is therefore important to gain insight in both the objective as well as the perceived environmental characteristics.

During the audits, neighbourhoods were observed systematically by two research assistants with a standardised scoring form that was developed specifically for screening Dutch neighbourhoods on environmental characteristics relating to children's physical activity behaviour [3]. The scoring form included the following topics: type of residences, outdoor play opportunities, sports facilities, public spaces, green and water, street pattern, traffic safety and area deprivation.

Although the scoring form was used and described before [36], this was the first time the measurement tool was used on a larger scale (33 neighbourhoods) and some improvements in the operation instructions were carried through to enhance reproducibility of the measurement tool. Firstly, the neighbourhood's boundaries were identified more explicitly and based on municipal information, which makes the outcomes of the observations easier to interpret and implemented by the local policy makers. Secondly, in stead of just walking through the neighbourhoods according to a route chosen by the observers during the audit, a random sample of 10% of all of the streets in one neighbourhood was selected for observation in advance (according to another Dutch neighbourhood observation protocol developed by Van Lenthe et al[37]). All remaining streets were observed whenever possible, considering that the mean duration of one neighbourhood observation was approximately 3 hours. Lastly, all observations were carried out during normal school days after school time and before dark, to mimic best the real conditions under which children are generally physically active in their neighbourhood.

In total, 33 neighbourhoods were selected for observation, covering a large part of the total study population (39% of parents and 38% of the children that filled in a questionnaire were living in one of the observed neighbourhoods, neighbourhoods were selected for audit only in case their residents were also included in the health survey). Because the neighbourhood observations were conducted between October and December 2008 (autumn), weather conditions were also monitored.

### Policy analysis

To map the local policy conditions in the four participating municipalities, a qualitative policy analysis was conducted. The aim of this policy analysis is to map the current multi-sector initiatives aimed at physical activity in children and to identify success and failure factors in developing and implementing multi-sector policies to stimulate physical activity in children. Although collaborations with private parties outside the municipal organisation (such as sports clubs or housing corporations) are very common and can also have beneficial effects on the integrated approach of stimulating physical activity, this was not taken into account; the policy analysis merely focuses on policy initiatives that involved more than one municipal policy sector.

Six policy sectors that have a potential influence on children's physical activity behaviour were selected: Public Health, Sports, Education & Youth, Spatial Planning, Traffic & Transport, and Safety. From each of these sectors, official policy documents (such as memoranda) were collected and screened on their content relating to the prevention of overweight or obesity, the stimulation of physical activity, or the influence on possible environmental determinants related to physical activity. Moreover, it was examined if the documents referred explicitly to physical activity in children and if the policy plans for promoting physical activity were mentioned to be from a multi-sector point of view (in other words, if other municipal sectors were involved in the development or realization of the policy plans). In total 29 policy documents were screened on their content.

In addition to the document analysis, one policy maker of each sector per city was interviewed using a semi-structured interview protocol including the following topics: network participants, collaboration structure and relations and critical success factors for multi-sector policy development. This yields more insight into the actual realization of the policy plans described in the policy documents and in the success and failure factors in multi-sector policy development as perceived by the actors themselves. Because the respondents were interviewed independently of each other, the viewpoints of different sectors could be identified. The policy analysis was conducted between February and May 2009.

### Interactive workshop session with local policy makers

In the development and implementation of policy plans and measures, scientific knowledge is only one source of information. Other sources of influence, such as experience and expertise, judgement, resources, values and political context, habits and tradition, lobbyists and pressure groups, may also play an important role in the policy development process [27]. Moreover, Armstrong et al. suggest that the utilisation of scientific knowledge is sector-specific and that the use of scientific research results is more common in the public health sector than in other sectors. Because this research project specifically addresses the opportunities for multi-sector policy measures, much emphasis is placed on the translation of the results of the first part of the project into concrete and feasible policy measures during the workshop sessions with policy makers.

The research described above will yield valuable insight into the environmental determinants of physical activity in children and into the current policy plans addressing these determinants. Also, insight is acquired regarding the critical success factors in the development and realization of multi-sector policy plans on a local level. In order to translate these research outcomes into concrete and feasible policy measures, each municipality is offered an semi-structured workshop session in which policy makers of different sectors participate. The workshop sessions with local policy makers are planned for 2009-2010 and will be set up in close collaboration with the local policy makers that are participating in these workshops.

To conclude this paragraph, table 5 gives an overview of the data collection in the project.

**Table 5:** Overview of data collection

	**Setting/Subjects**	**Period**	**Goal**	**Methods**
Part 1: Health survey	6,601 parents and 3,449 children of 42 primary schools in four cities	September 2007 - January 2008	Obtain information about perceived environmental characteristics in relation to children's physical activity level.	- Questionnaires for parents - Questionnaires for children - Questionnaires for school management
Part 2: Neighbourhood audits	33 neighbourhoods in four cities	October 2008 - December 2008	Obtain objective data on neighbourhood characteristics in relation to children's physical activity level.	- Neighbourhood audits using a standardised observation protocol
Part 3: Policy analysis	29 policy documents and interviews with policy makers from six different policy sectors in four cities	February 2009 - May 009	Obtain an overview of current policy situation and success and failure factors in the development of multi-sector policies.	- Document analysis of six different policy sectors - Interviews with local policy makers of six different policy sectors
Part 4: Workshop sessions	policy makers from six different policy sectors in four cities	2009-2010	Assess feasibility of concrete multi-sector policy measures on an local level that enhance physical activity in children	Interactive workshop sessions with local policy makers of six different policy sectors

### Statistical analysis

The quantitative data obtained by the questionnaires and neighbourhood observations will be analysed using standard statistical software packages (SPSS) and software packages suitable for multi level analysis to adjust for clustering within the data set (SAS, MlWin). The qualitative data obtained by the policy analysis and interactive workshop sessions will be analysed using software for qualitative analysis (ATLAS.ti).

## Discussion

This paper describes the study design of a project that focuses on establishing multi-sector policies that stimulate physical activity in children. Next to extensive research on the environmental determinants of physical activity in children, much emphasis is placed on translation of the research outcomes into clear cut policy measures that are tailored to the needs and possibilities of local policy makers.

The combination of quantitative and qualitative research provides a strong basis for the development of evidence based policy plans. Not only the actual contribution of environmental characteristics to children's physical activity level is examined, also the practical implications and limitations in local policy development are taken into account.

Considering the data collection within this project, several strong points can be mentioned. Firstly, the combination of subjective as well as objective measurement of neighbourhood characteristics by means of questionnaires and neighbourhood audits contributes to a better understanding of the role of environmental characteristics in the physical activity level of children. Secondly, school environment and school policies regarding physical activity were examined separately by means of a questionnaire for the school management. Thirdly, this is one of the first studies that addresses children's own perception of their living environment related to their physical activity behaviour. Especially for older children, this yields valuable insight additional to the perceptions of the parents. Further, the research addresses physical as well as social environmental characteristics, which stimulates a multi-sector approach (policy sectors involved with physical neighbourhood characteristic such as Spatial Planning or Traffic & Transport and policy sectors involved with social neighbourhood characteristics such as Safety or Education & Youth). In addition, the large scale set up of the study, the high response rates and the great variance in environmental characteristics of the schools and neighbourhoods included in the study all contribute to the quality of the data. Lastly, much emphasis is placed on the interaction with policy makers at the local level to enhance translation of the research outcomes into concrete policy measures.

Possible limitations of the project lie in the fact that questions about the perceived environmental characteristics in the questionnaires that were used, were not validated, but only pretested in the target population. Validation of the perceived environment is very difficult, because there is no "golden standard" against which to compare a perceived measure. It can even be argued that the perceived measure is the reality [9]. Another limitation of the study is that the data from the questionnaires on physical activity and BMI are based on (parental) self report data, which is known to be less accurate and reliable than more objective measurements such as accelerometry and height and weight measurements by trained researchers. Due to practical limitations, it was not possible to asses physical activity and BMI in a more objective way. Lastly, because the cross-sectional nature of the study, no causal relationships can be demonstrated.

Multi-sector policy measures aimed at making neighbourhoods more active-friendly are promising tools in structurally tackling inactivity among youth. There is an urgent need for studies that support (local) policy makers in the development and realization of such policy plans. Besides the scientific underpinning of possible policy measures, attention should be drawn to the practical feasibility of policy measures.

## Competing interests

The authors declare that they have no competing interests.

## Authors' contributions

JS wrote the research proposal which was approved by The Netherlands Organisation for Health research and Development (ZonMw) for funding. MJA conducted the data collection and drafted the manuscript. JS, HvO and IvdG supervised the data collection and reviewed the manuscript. All authors read and approved the final manuscript.

## Pre-publication history

The pre-publication history for this paper can be accessed here:


